# LABFNet: A Restoration Network Guided by the LAB Colour Space and Frequency-Domain Constraints

**DOI:** 10.3390/jimaging12070332

**Published:** 2026-07-22

**Authors:** Yaqian Zhang, Guanjun Wang, Quan Zhang, Bochao Zhou

**Affiliations:** 1 School of Information and Communication, Hainan University, Haikou 570028, China; 993888@hainanu.edu.cn; 2 School of Electronic Science and Engineering, Hainan University, Haikou 570028, China; wangguanjun@hainanu.edu.cn; 3 School of Information and Communication Engineering, North University of China, Taiyuan 030051, China; 4 Algorithm Department, Xi’an Jiaying Intelligent Technology Co., Ltd., Xi’an 710000, China

**Keywords:** mural images, laboratory frequency network, spatial-domain features, frequency consistency

## Abstract

In the restoration of mural images with rich colour information and complex texture structures, existing techniques typically extract the spatial-domain features in the Red–Green–Blue (RGB) colour space. However, the three RGB channels are physically decoupled without unified perceptual colour correlation constraints, which often leads to noticeable colour deviation in damaged regions with large colour variations. In addition, restoring both high-frequency texture details and low-frequency global structures in a mixed-frequency spatial domain can create conflicts between frequencies, making it difficult to generate realistic high-frequency details. To address these issues, we propose the laboratory frequency network (LABFNet), a restoration network guided by the laboratory (LAB) colour space and frequency-domain constraints. Our model has two key improvements: (1) it incorporates colour parameters from the LAB space to model colour loss in murals, and (2) it decomposes the image into low- and high-frequency components and enforces frequency consistency during restoration. In the Dunhuang 20–40% mask-ratio setting, the Peak Signal-to-Noise Ratio (PSNR) and Structural Similarity Index (SSIM) improved by 1.58% and 0.27%, respectively, while the Mean Absolute Error (MAE), Learned Perceptual Image Patch Similarity (LPIPS) and CIEDE2000 decreased by 5.87%, 6.5%, and 21.65%, respectively. Experimental results on benchmark datasets show that LABFNet reduces colour deviation and structural defects.

## 1. Introduction

As an invaluable cultural heritage, murals are of great importance for studying ancient social life and historical change. However, environmental factors and human activities have caused varying degrees of damage, including cracking, flaking, and fading [1]. Manual restoration remains the primary approach, but it requires specialist expertise and the process is irreversible [2]. Comparatively speaking, digital restoration technology has therefore attracted sustained attention because it is non-destructive, efficient, and reversible [3]. With the rapid development of artificial intelligence and deep learning, many high-performing models have emerged in image restoration, which provide potential solutions for mural restoration [4,5]. Early methods were mainly based on Convolutional Neural Network (CNN) and U-shaped Network (U-Net) architectures [6,7], but their limited receptive fields made it difficult to capture long-range dependencies across distant image regions [8,9,10]. This led to the introduction of contextual mechanisms, which produced more refined texture features [11,12]. Subsequently, generative adversarial network (GAN)-based methods offered a new direction for image restoration [13,14]. However, the generative loss and adversarial loss emphasise different objectives: adversarial loss tends to favour high-frequency texture information, whereas reconstruction loss focuses more on low-frequency global structure. This conflict can hinder the generation of realistic high-frequency details [15,16,17]. More recently, Transformer-based models have attracted considerable attention because they support global long-range modelling and have shown strong performance in image restoration [18,19,20,21]. Even so, high computational cost and insufficient recovery of high-frequency detail remain important challenges [22,23,24]. Diffusion models have also shown promise, but because they are designed to take pure Gaussian noise as input rather than incomplete images containing useful contextual information, they may generate content that is inconsistent with the surrounding context [25,26].

**Figure 1:**
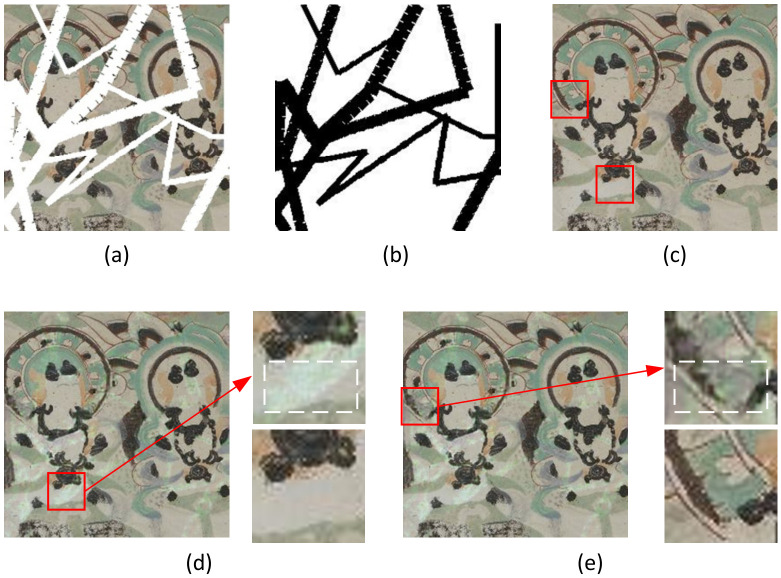
Colour deviation and unrealistic high-frequency detail artefacts in mural restoration. (**a**) Corrupted image; (**b**) mask; (**c**) ground truth; (**d**) colour-deviation artefacts; (**e**) unrealistic high-frequency detail artefacts. Red boxes highlight the main differences. In subfigures (**d**) and (**e**), the right halves present zoomed-in patches of the reconstructed image and the ground-truth image, respectively. Regions with prominent discrepancies are marked by white dashed boxes.

Although these methods perform well in image restoration, most still extract spatial-domain features in the RGB colour space. However, mural images contain not only complex texture structures but also rich colour information. The three RGB channels store decoupled light intensity information without built-in chromatic correlation constraints; convolution and upsampling operations can easily introduce channel misalignment, ultimately leading to serious colour deviation [27], as shown in Figure 1d. In addition, the spatial domain contains both high-frequency texture information and low-frequency global structure, which makes it difficult to maintain frequency consistency during restoration and therefore generate realistic high-frequency details [28], as shown in Figure 1e.

RGB channels correspond to physical light intensity without uniform perceptual colour distance mapping; channel-independent convolution and upsampling perform feature mapping without constraining chromatic correlation, resulting in unconstrained chromatic offset in damaged areas with drastic colour mutation. By contrast, the LAB colour space is perceived as uniform to the human visual system: the Euclidean distance between two LAB pixel points linearly matches the human-perceived colour difference. RGB channel operations decouple luminance and chrominance, while LAB separates luminance L and two chromatic axes A/B, enabling independent regularisation of structural brightness and colour offset, which directly suppresses cross-channel misalignment artefacts.

When image restoration is executed entirely within the spatial domain, low-frequency global mural contours and high-frequency fine crack textures are mixed in the same feature map. Unseparated spatial signals couple low-frequency global contours and high-frequency tiny textures into one feature tensor, and a single loss function cannot assign differentiated optimisation weights to two frequency components. Our frequency decomposition strategy splits the image into independent low- and high-frequency sub-signals via Gaussian filtering, applying separate L1 constraints to decouple the two optimisation objectives; FFT frequency spectrum loss further regularises the global spectral distribution to eliminate frequency inconsistency conflicts fundamentally.

To address colour distortion artefacts, Meng Wu et al. incorporated LAB colour parameters to characterise mural chromatic information [29]. However, their pipeline requires high-resolution multi-lens Tang tomb mural images captured via a camera array, a condition barely satisfied by publicly available mural datasets. Structure flow constructs a pigment spectral library through large-scale pigment spectral analysis, and leverages a spatially guided gated generative adversarial network for mural colour restoration [30]. In terms of frequency consistency regularisation, LaMa [31] integrates frequency-domain representations, yet only targets the limited receptive field dilemma under large mask ratios, without dedicated constraints to harmonise frequency distributions. WaveFill [32] decomposes images into low- and high-frequency sub-bands via wavelet transformation and applies distinct processing branches to balance frequency consistency. Nevertheless, its core motivation lies in mitigating conflicting optimisation objectives between generator and discriminator modules within GAN frameworks, and the model is primarily optimised for natural images rather than specialised mural restoration tasks.

To address these limitations, we propose LABFNet, a restoration model guided by LAB colour parameters and frequency-domain constraints. By introducing LAB-space colour parameters, our framework strengthens colour correlation across channels and reduces colour deviation. In addition, frequency-domain constraints are used to regulate frequency consistency and generate more realistic high-frequency details. The main contributions of this work are as follows:

1. We propose LABFNet, a novel mural-restoration model that incorporates LAB colour parameters and frequency-domain constraints to reduce colour deviation and improve frequency consistency during restoration.
2. We introduce LAB colour-space parameters by converting mural images from RGB to LAB, thereby strengthening inter-channel colour correlation. Information from the A and B channels is used to guide colour restoration during image generation.
3. We introduce frequency-domain constraints by decomposing mural images into low- and high-frequency components and enforcing frequency consistency during restoration, thereby guiding the generation of more realistic high-frequency details.

Extensive experiments show that, compared with state-of-the-art restoration models, LABFNet reduces colour deviation and produces more realistic high-frequency details. The remainder of this paper is organised as follows: Section 2 describes the proposed method in detail; Section 3 presents the experimental results; Section 4 presents the discussion; and Section 5 concludes the paper.

## 2. Methods

### 2.1. Overview

**Figure 2:**
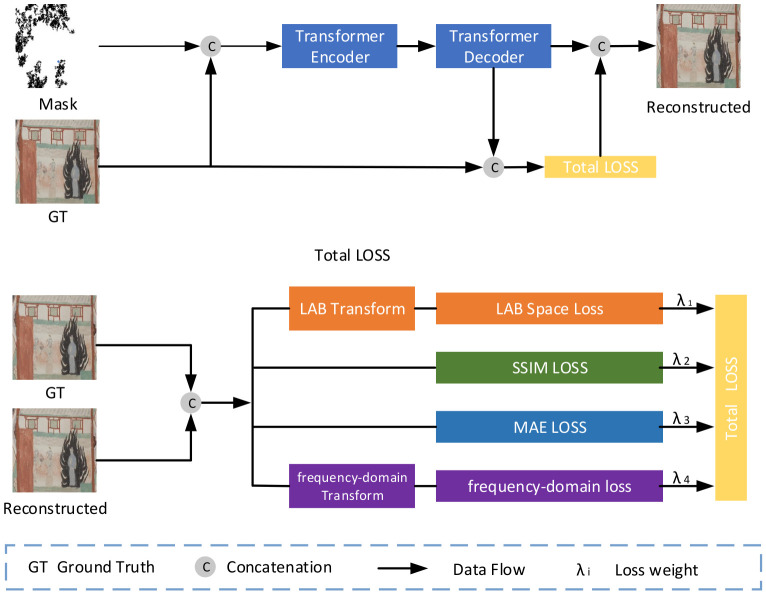
Overview of the proposed LABFNet framework. The model is built upon CMAMR. To address CMAMR’s limitations in colour deviation and frequency consistency, we introduce LAB loss and frequency-domain loss. The upper part shows the overall architecture and the lower part shows the loss design.

The proposed restoration network is shown in Figure 2. Inspired by Contextual Mask-Aware Mural Restoration (CMAMR) [33], our backbone adopts a similar architecture. To address the limitations of CMAMR in colour deviation and frequency consistency, we introduce LAB-space colour parameters to correct colour deviation. In addition, we impose constraints in both the spatial and frequency domains to regulate frequency consistency and generate more realistic high-frequency details during restoration.

The complete forward procedural flow is divided into four sequential steps: first, the input masked mural image passes backbone feature extraction to generate a preliminary restored RGB image; second, this image and the ground truth (GT) are converted to LAB colour space to calculate LAB loss; then, the restored and ground-truth images are decomposed into low/high-frequency components via Gaussian filtering, and then transformed to the frequency domain via FFT to calculate frequency-domain (Fre) loss; and finally, LAB loss, Fre loss, global SSIM loss, and pixel MSE loss are aggregated into total loss, where four weights $\lambda_{1}$, $\lambda_{2}$, $\lambda_{3}$, and $\lambda_{4}$ are dynamically learned via homoscedastic uncertainty.

### 2.2. LAB Space Loss

The RGB colour space is widely used because of its hardware compatibility, but in mural restoration, it is prone to colour deviation. By contrast, the LAB colour space better reflects perceptual colour differences: CIE Lab is a perceptually uniform colour space, where the Euclidean distance between two pixel points can roughly approximate human visual colour differences; therefore, we adopt channel MSE as the differentiable training loss for network backpropagation, and utilise CIEDE2000 (DeltaE00) as an independent offline quantitative evaluation metric to strictly measure real human-perceived colour deviation. We therefore introduce LAB-space terms into the loss function to reduce colour deviation. Specifically, in LAB space, the L channel represents luminance information and is mainly related to structural and edge details, whereas the A and B channels represent chromatic information [34]. Accordingly, SSIM is applied to the L channel to measure structural consistency, while MSE is applied to the A and B channels to measure chromatic error. In addition, because colour deviation tends to be concentrated in damaged regions, we use the Euclidean distance between each damaged pixel and its nearest non-damaged pixel as a weighting factor in the damaged-region mean squared error (MSE). MSE is then computed separately for damaged and non-damaged regions.

Notably, the Dunhuang and MuralDH datasets adopted in our experiments are publicly accessible, and all input images conform to the sRGB colour space. The sRGB-to-CIE Lab conversion is implemented via standard CIE formulas, with D65 set as the reference illuminant [35]. Images in the two datasets were captured under unregulated natural lighting without colour calibration. If mural data were to be collected under controlled illumination and calibrated with standard colour charts in subsequent research, ambient-light-induced colour bias can be largely eliminated. This enables the proposed LAB loss to concentrate on fitting authentic pigment fading patterns instead of lighting interference, further improving the perceptual colour consistency of restored mural results.

The above process can be formulated as follows:

(1) $${Loss}_{LAB}=SSIM\left({res}_{L},{tar}_{L}\right)+{MSE}_{p}\left({res}_{AB},{tar}_{AB}\right)+{MSE}_{w}\left({res}_{AB},{tar}_{AB}\right)$$

where ${Loss}_{LAB}$ denotes the LAB-space loss; res and tar denote the restored image and the ground-truth image, respectively; $SSIM\left({res}_{L},{tar}_{L}\right)$ denotes SSIM computed on the L channel; ${MSE}_{p}\left({res}_{AB},{tar}_{AB}\right)$ denotes MSE computed on the non-damaged regions of the A and B channels; and ${MSE}_{w}\left({res}_{AB},{tar}_{AB}\right)$ denotes the distance-weighted MSE computed on the damaged regions of the A and B channels. The weighted MSE is defined as follows:

(2) $${MSE}_{w}\left({res}_{LAB},{tar}_{LAB}\right)=\frac{1}{n}\sum_{i=1}^{n}\frac{1}{d}(x_{i}-{\hat{x}}_{i}{)}^{2}$$

where n denotes the number of pixels in the damaged region, and d denotes the 2D spatial geometric Euclidean pixel distance between each damaged pixel and its nearest intact pixel on the image plane. The term $\frac{1}{d}$ assigns higher weights to pixels closer to the damaged boundaries, thereby facilitating smoother edge transitions. $x_{i}$ and ${\hat{x}}_{i}$ denote the pixel values at the corresponding positions in the restored image and the ground-truth image, respectively.

### 2.3. Frequency-Domain Loss

Most existing image restoration models are primarily based on spatial-domain features, but such features still suffer from limitations in regulating frequency consistency and capturing global structures. By contrast, frequency-domain features are more effective for modelling global structures and generating more realistic high-frequency details [36]. Motivated by frequency domain image translation (FDIT) [37], we first decompose the image into high- and low-frequency components using Gaussian filtering, and compute the corresponding losses separately to enforce frequency consistency. Subsequently, the Fast Fourier Transform (FFT) is applied to map the image from the spatial domain to the frequency domain, where additional frequency-domain loss terms are introduced to better capture global structural information. The above process can be formulated as follows:

(3) $${Loss}_{fre}=||{res}_{Low}-{tar}_{Low}|{|}_{1}+||{res}_{High}-{tar}_{High}|{|}_{1}+|\left|F_{res}^{R}-F_{tar}^{R}\right|{|}_{1}$$

where ${Loss}_{fre}$ denotes the frequency-domain loss; $∥\;{res}_{Low}-{tar}_{Low}\;{∥}_{1}$ denotes the $L_{1}$ distance between the low-frequency components and $||{res}_{High}-{tar}_{High}|{|}_{1}$ denotes that between the high-frequency components; ${res}_{Low}$ and ${res}_{High}$ denote the low- and high-frequency components of the restored image, respectively; ${tar}_{Low}$ and ${tar}_{High}$ denote the corresponding components of the ground-truth image; and $F_{res}^{R}$ and $F_{tar}^{R}$ denote the real-valued frequency-domain representations of the restored and ground-truth images, respectively.

The low- and high-frequency components are computed as follows:

(4) $$X_{Low}\left(i,j\right)=\sum_{m=-k}^{k}\sum_{n=-k}^{k}X\left(i+m,j+n\right).*\frac{1}{2\pi \sigma^{2}}e^{-\frac{m^{2}+n^{2}}{2\sigma^{2}}}$$

(5) $$X_{High}\left(i,j\right)=X\left(i,j\right)-X_{Low}\left(i,j\right)$$

where $X_{Low}$ and $X_{High}$ denote the low- and high-frequency components of the image, respectively; X denotes the original image; σ denotes the standard deviation; and k denotes the Gaussian-kernel radius. m and n denote independent dummy variables for Gaussian-kernel neighbourhood sliding, ranging from −k to k, which implement spatial convolution by traversing all local pixels centred at coordinate $\left(i,j\right)$.

The frequency-domain mapping and the conversion from complex-valued representations to real-valued forms are defined as follows:

(6) $$F\left(u,v\right)=\sum_{x=0}^{M-1}\sum_{y=0}^{N-1}f\left(x,y\right).*\;e^{-j2\pi \left(\frac{u.*x}{M}+\frac{v.*y}{N}\right)}$$

(7) $$F^{R}\left(u,v\right)=\log\left(1+\sqrt{(ReF\left(u,v\right){)}^{2}}+\sqrt{(ImF\left(u,v\right){)}^{2}}\right)+\epsilon$$

where $F\left(u,v\right)$ denotes the frequency-domain representation obtained from the spatial-domain image f(x,y); M and N denote image width and height; $F^{R}(u,v)$ denotes the real-valued magnitude representation derived from the complex spectrum; ReF and ImF denote the real and imaginary parts of the frequency-domain data, respectively; and ε is a small constant added for numerical stability to avoid zero values.

The Gaussian-filtering-based low/high-frequency decomposition and subsequent FFT spectrum transformation are implemented independently on all three RGB colour channels of the input image. Each channel is split into its corresponding low- and high-frequency components separately, and L1 frequency loss is calculated channel-wise and then averaged across three channels to form the final frequency loss term.

### 2.4. Overall Loss

Following the homoscedastic uncertainty multi-task loss weighting theory proposed by Kendall et al. [38], we treat each loss term as an independent regression task with task-specific uncertainty noise $\sigma_{i}$. The multi-task loss can be mathematically modelled as

(8) $$L_{total}=\frac{1}{2\sigma_{1}^{2}}{Loss}_{LAB}+\frac{1}{2\sigma_{2}^{2}}{Loss}_{fre}+\frac{1}{2\sigma_{3}^{2}}SSIM\left(res,tar\right)+\frac{1}{2\sigma_{4}^{2}}MSE\left(res,tar\right)+\sum_{i=1}^{4}\log\left(\sigma_{i}\right)$$

where $\sigma_{1}$, $\sigma_{2}$, $\sigma_{3}$, and $\sigma_{4}$ are four trainable uncertainty parameters optimised together with network weights during backpropagation. res denotes the restored image, tar denotes the ground-truth image, SSIM(res,tar) denotes structural similarity, and MSE(res,tar) denotes mean squared error.

The logarithmic regularisation term avoids infinite weight values caused by vanishing uncertainty. When the noise σ increases, the corresponding weight will decrease; on the other hand, as it decreases, the corresponding weight will increase, to self-optimise the overall objective function.

Let $\frac{1}{2\sigma_{i}^{2}}=\lambda_{i}$; then, the overall loss function of the proposed model can be simplified as

(9) $$L_{total}=\lambda_{1}{Loss}_{LAB}+{\lambda_{2}Loss}_{fre}+\lambda_{3}SSIM\left(res,tar\right)+\lambda_{4}MSE\left(res,tar\right)$$

Compared with the traditional trial-and-error fixed weight combination, this adaptive weighting mechanism has two core advantages:

(1) Dynamic task balance: Different loss terms have disparate value magnitudes and optimisation convergence speeds. Fixed manual weights cannot adapt to training stage changes; homoscedastic uncertainty automatically assigns larger weights to loss branches with higher reconstruction uncertainty at each iteration, dynamically balancing colour restoration, frequency consistency, and spatial reconstruction objectives without manual hyperparameter tuning.
(2) Objective-driven optimisation: The weight learning process is fully data-driven rather than artificially predefined, eliminating arbitrary loss combination bias, ensuring the overall objective function always optimises the mural restoration task from the joint perspective of colour perception, frequency features, and pixel reconstruction.

The calculation sequence of total loss follows a branch-parallel aggregation mechanism: the LAB colour loss and frequency-domain loss branches are independently computed after image restoration, and then fused with global spatial reconstruction metrics SSIM and MSE. Each loss term is assigned an automatically optimised weight without manual tuning, and the final total loss backpropagates to update all network parameters synchronously.

### 2.5. Compared Methods

Multi-level Interactive Siamese Filtering (MISF) [39] and Hypernetwork Instruction Tuning (HINT) have served as classic high-quality restoration models in the field of mural restoration in recent years, and both take the Dunhuang mural dataset as the verification object. In addition, although Meng Wu et al. also adopted the LAB space to eliminate colour deviation, their work specifically targets Tang tomb murals, which differ substantially from Dunhuang murals due to distinct preservation environments and erosion modes. While the large mask inpainting (LaMa) model also leverages frequency-domain processing, its effectiveness is only validated on the Places and CelebAHQ datasets, and the literature does not explicitly verify whether it performs equally well on mural datasets. Mural datasets possess clear distinctions from Places and CelebAHQ, such as richer colour information and elaborate texture details. Finally, since the proposed LABFNet is improved on the basis of the CMAMRmodel, CMAMR, MISF, and HINT are selected as benchmark models for fair comparison. All models are retrained from scratch under identical experimental conditions using the MuralDH and Dunhuang datasets to guarantee the fairness of quantitative evaluation.

### 2.6. Evaluation Metrics

We adopt multiple complementary metrics for comprehensive performance evaluation. PSNR and MAE measure pixel reconstruction fidelity for global mural structure restoration; SSIM evaluates overall structural similarity; LPIPS quantifies human perceptual high-frequency texture realism to verify the effect of frequency-domain loss on crack detail generation; and CIEDE2000 exclusively quantifies perceptual colour deviation to validate the colour correction performance of LAB colour space loss. All the indicators collectively support our two core technological innovations, namely the LAB colour constraint and the frequency consistency constraint.

## 3. Results

In this study, we conducted extensive evaluations of the proposed method. First, this section describes the experimental settings, including the datasets and implementation details. Subsequently, LABFNet is compared with state-of-the-art methods, and both quantitative and qualitative results are presented to demonstrate the superiority of the proposed approach. Ablation studies were also performed to verify the necessity of each key component.

### 3.1. Experimental Settings

#### 3.1.1. Datasets

To evaluate the proposed model and compare it with other methods, we adopted two open access mainstream datasets in the mural restoration field: (1) MuralDH [40]: a Dunhuang mural dataset containing 961 high-resolution images (760 for training and 201 for testing), with pixel-level damage annotations and corresponding degradation masks; (2) Dunhuang [41]: a Dunhuang grotto mural dataset consisting of 600 mural images (500 for training and 100 for testing) together with corresponding degradation masks.

#### 3.1.2. Implementation Details

The proposed model was implemented in PyTorch 2.7.1 and trained on an NVIDIA RTX 5090 GPU using 256 × 256 input images for more than 1000 epochs. The AdamW optimiser [42] was used with an initial learning rate of 0.0002, together with a cosine-annealing scheduler [43]. To improve model robustness, standard data augmentation techniques are applied, including random cropping, flipping, and rotation. For 256 × 256 input images, the Gaussian filter kernel size was set to 21, consistent with FDIT.

### 3.2. Quantitative Evaluation

We conducted extensive quantitative evaluations on both the MuralDH and Dunhuang datasets. For the MuralDH dataset, the provided standard masks were directly used, whereas for the Dunhuang dataset, standard masks with mask ratios ranging from 0% to 20% were selected from the provided masks, and additional random masks with ratios of 20–40% were introduced to evaluate model performance under more challenging conditions. For a fair comparison, all experiments were conducted using the officially released pre-trained models on the corresponding datasets. It is worth noting that our overall loss function jointly incorporates SSIM and MSE loss terms, which the network directly minimises during the training phase. Accordingly, the performance superiority of LABFNet on SSIM and MAE metrics is partially predictable, as the model is explicitly optimised toward these two pixel-level reconstruction objectives. To avoid overstating the model’s advantages, relying on training-targeted metrics, we place more emphasis on analysing LPIPS, a perceptual metric excluded from our loss function and serving as an unbiased indicator for visual authenticity evaluation. The quantitative results are presented in Table 1 and Table 2, where LABFNet achieves superior overall performance compared with existing methods. Compared with the previous state-of-the-art method CMAMR, our approach improved PSNR and SSIM by 1.27% and 0.25%, respectively, while reducing MAE, LPIPS, and CIEDE2000 by 2.06%, 6.5%, and 33.5% on the MuralDH dataset, respectively. On the Dunhuang dataset with standard masks, PSNR and SSIM improved by 1.41% and 0.27%, while MAE, LPIPS, and CIEDE2000 decreased by 4.77%, 4.5%, and 37.7%, respectively. Under the more challenging 20–40% mask-ratio setting on the Dunhuang dataset, PSNR and SSIM improved by 1.58% and 0.27%, while MAE, LPIPS, and CIEDE2000 decreased by 5.87%, 6.5%, and 21.65%, respectively. Higher PSNR and SSIM indicate better reconstruction fidelity and structural similarity, whereas lower MAE and LPIPS values indicate lower global pixel-level reconstruction errors and better visual perceptual consistency between restored images and ground-truth references, respectively. Lower CIEDE2000 values correspond to slighter colour discrepancies between reconstructed images and the ground truth. The consistent improvements across different datasets and mask ratios demonstrate the strong generalisation capability of the proposed method.

### 3.3. Qualitative Evaluation

**Figure 3:**
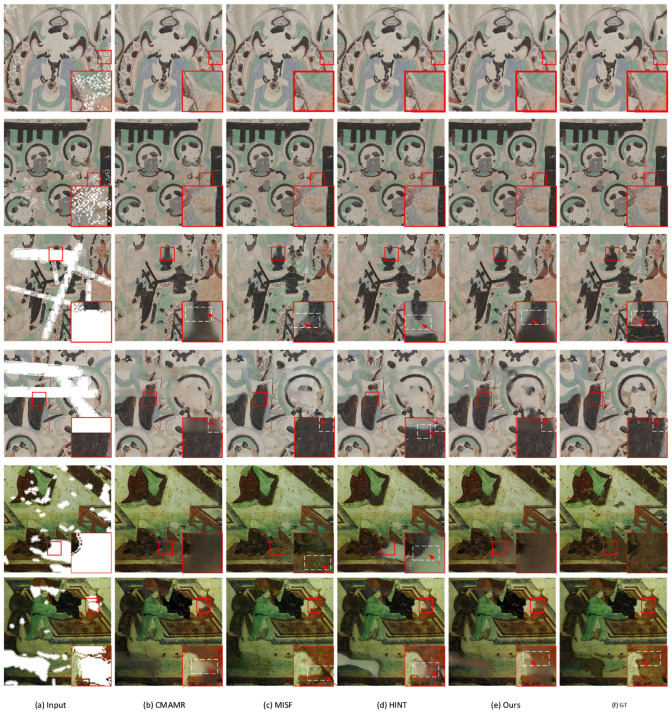
Qualitative comparison with state-of-the-art methods. From left to right: (**a**) masked input, (**b**) CMAMR [33], (**c**) MISF [39], (**d**) HINT [19], (**e**) our method, and (**f**) ground truth (GT). The top two rows correspond to the Dunhuang dataset with mask ratios of 0–20%, the middle two rows correspond to the same for 20–40%, and the bottom two rows correspond to the MuralDH dataset. The main differences are highlighted using red rectangles. Regions with prominent discrepancies are marked by white dashed boxes and red arrows.

The visual comparisons shown in Figure 3 further validate the effectiveness of the proposed method. Although a perfectly faithful restoration of degraded murals remains challenging, LABFNet shows a stronger ability to preserve plausible textures and structural details. When the mask region is small, all methods perform well, but as the mask region increases, CMAMR exhibits clear boundary blurring, MISF preserves structure relatively well but introduces noticeable colour deviation, and HINT tends to generate unrealistic high-frequency details. By contrast, our method produces more plausible high-frequency details and better overall colour consistency. In particular, it handles complex crack patterns and severe degradation more effectively, producing restorations that are more visually coherent and contextually consistent.

### 3.4. Ablation Study

Our proposed LABFNet is improved based on the CMAMR model, which means the network backbone architecture follows the same structure as CMAMR, and our core improvements concentrate on redesigning the loss function of CMAMR. The ablation experiments verifying the effectiveness of the CMAMR backbone architecture were fully completed and discussed in the original CMAMR work. Therefore, the ablation experiments in this study mainly focus on validating the effectiveness of our newly designed loss function modules.

**Figure 4:**
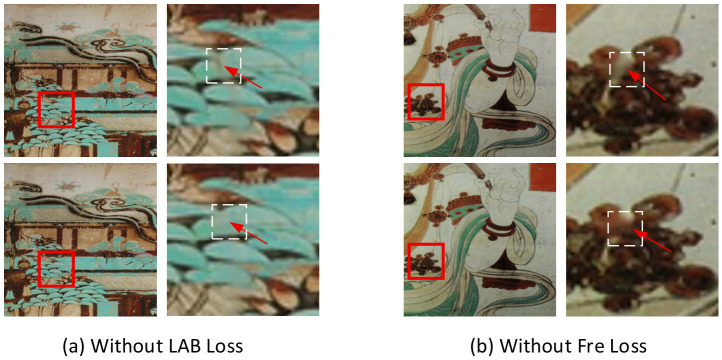
(**a**) and (**b**) present the ablation results of the LAB loss and the frequency-domain loss, respectively. In each example, the upper row shows the results without the corresponding module. For clearer visualisation, selected regions are enlarged in each ablation experiment. Regions with prominent discrepancies are marked by white dashed boxes and red arrows.

To verify the effectiveness of the key components, ablation studies were conducted on the MuralDH dataset. Specifically, the LAB loss and Fre loss modules were individually removed from the model for analysis. To provide a clearer comparison, representative images were selected for each ablation setting, as illustrated in Figure 4, and the results show that each component plays an important role in the proposed network. The LAB module helps preserve colour consistency, while the frequency-domain loss helps generate more realistic high-frequency details. The quantitative results reported in Table 3 further support this analysis. Removing the LAB loss leads to elevated MAE, LPIPS, and CIEDE2000 values, which is attributed to the relatively large proportion of regions with colour distortion compared to texture-structured areas. MAE, which quantifies global pixel-wise deviation, increases significantly. Meanwhile, chromatic offsets induce misalignment between multi-layer convolutional feature vectors of ground-truth and restored images, raising LPIPS scores. As CIEDE2000 is designed specifically to measure colour differences, colour distortion also causes a substantial degradation in this metric. Eliminating the frequency-domain loss results in lower PSNR and SSIM. Structural distortions introduce severe pixel misalignment errors, even though textured regions occupy a smaller area than colour-biased regions do. The squared operation embedded in the PSNR calculation further amplifies such positional errors, degrading PSNR performance. Since SSIM evaluates structural similarity, structural artefacts inevitably lead to a notable drop in SSIM values. When both modules are integrated, the full model achieves the best performance across all evaluation metrics, demonstrating the complementary effects of the LAB loss and the frequency-domain loss.

## 4. Discussion

This study proposes LABFNet, a mural restoration framework constrained by the LAB colour space and frequency-domain regularisation, to address two critical artefacts prevalent in deep learning-based mural inpainting: chromatic distortion induced by decoupled RGB channels, and unnatural high-frequency textures arising from conflicting optimisation of mixed-frequency features.

We first compare LABFNet with representative state-of-the-art mural restoration models. MISF prioritises high-fidelity restoration via semantic and image-level filling strategies, while HINT leverages the long-range modelling capacity of Transformer architectures. CMAMR, by contrast, maintains comprehensive mask guidance throughout the pipeline and efficiently captures multi-scale contextual information. Despite their competitive performance, none of these models is specially optimised for the two core defects of colour distortion and frequency inconsistency. Built upon the CMAMR backbone, our proposed method incorporates LAB colour parameters and frequency consistency constraints to mitigate the above issues, delivering superior restoration performance.

We further elaborate on the underlying mechanism of the two core loss branches. First, we analyse the rationality of adopting LAB loss instead of a standalone RGB reconstruction loss. Convolution and upsampling operations on standard RGB inputs readily trigger cross-channel misalignment, which yields severe colour deviation within damaged mural regions. Incorporating LAB colour parameters strengthens chromatic correlations across channels and effectively suppresses such colour distortion. Second, we interpret the performance gains brought by frequency-domain loss. Conventional mural restoration models process low-frequency global contours and high-frequency crack textures within identical feature maps, creating conflicting optimisation objectives that degrade the authenticity of fine textures. By decomposing mural images into low- and high-frequency components and imposing frequency consistency constraints during reconstruction, our approach guides the network to generate more realistic high-frequency details.

Quantitative and qualitative experimental results on the MuralDH and Dunhuang datasets consistently validate the superiority of LABFNet over all comparative baselines. Nevertheless, the framework still has a clear limitation: slight blurry artefacts remain at the boundaries of large damaged mural regions, which impairs the continuity of edge colour and texture.

## 5. Conclusions

In this work, we developed LABFNet, a dedicated mural inpainting network equipped with LAB chromatic regularisation and frequency-domain constraints, to resolve the two dominant defects of existing mural restoration models, namely, RGB-induced colour distortion and unnatural high-frequency textures caused by mixed-frequency optimisation conflicts. Based on the CMAMR backbone, the introduced LAB loss strengthens cross-channel chromatic correlation to reduce colour deviation, while the frequency decomposition loss decouples global structural and local texture optimisation for more natural crack details. Sufficient experiments on two mural datasets demonstrate that LABFNet achieves consistent quantitative and qualitative improvements compared with MISF, HINT, and CMAMR.

To address the boundary blurring limitation observed in large damaged areas, we outline three clear directions for future research: We plan to design a learnable boundary weight mask branch to dynamically strengthen loss constraints at the junction of damaged and intact regions, optimising the edge colour transition and texture continuity without introducing additional computational overhead. We intend to integrate multi-temporal mural images captured under varying lighting conditions, embed a temporal feature alignment branch into LABFNet, and introduce inter-frame temporal consistency loss. Redundant multi-frame information will relieve boundary blur for single damaged samples and improve restoration robustness against uneven illumination degradation. We will explore cross-modal fusion by building an encoder to combine 2D RGB mural images with LiDAR point cloud and 3D depth scan data. Additional depth-aware frequency constraints will distinguish texture fading from geometric flaking damage, enabling joint restoration of mural colour distortion and three-dimensional structural defects.

No generative artificial intelligence tools were used to generate any model architectures, experimental data, or core research conclusions of this paper.

## Figures and Tables

**Table 1:** Quantitative comparison results (Mean ± SD) on the Dunhuang dataset with current mainstream methods, with mask ratios set at 0–20% and 20–40%. The best results are marked in bold and the second-best results are underlined.

Dunhuang		CMAMR	MISF	HINT	Ours
**0–20%**	**PSNR ↑**	43.8476 ± 1.2569	40.6103 ± 1.7135	43.4013 ± 1.1374	**44.4646 ± 1.2303**
	**SSIM ↑**	0.9931 ± 0.0012	0.9896 ± 0.0069	0.9923 ± 0.0015	**0.9952 ± 0.0011**
	**MAE ↓**	0.3139 ± 0.0856	0.4504 ± 0.1067	0.3382 ± 0.0639	**0.2996 ± 0.0811**
	**LPIPS ↓**	0.0069 ± 0.0017	0.0095 ± 0.0034	0.0072 ± 0.0018	**0.0066 ± 0.0016**
	**CIEDE2000 ↓**	0.1788 ± 0.0414	2.3348 ± 0.1632	0.1590 ± 0.0284	**0.1298 ± 0.0265**
**20–40%**	**PSNR ↑**	30.0332 ± 0.8567	29.0854 ± 0.6128	30.2370 ± 0.8643	**30.7143 ± 0.8463**
	**SSIM ↑**	0.9294 ± 0.0077	0.9116 ± 0.0085	0.9306 ± 0.0078	**0.9331 ± 0.0074**
	**MAE ↓**	2.5729 ± 0.2906	3.2554 ± 0.3472	2.4732 ± 0.2695	**2.3361 ± 0.2685**
	**LPIPS ↓**	0.1020 ± 0.0098	0.1753 ± 0.0075	0.1025 ± 0.0078	**0.0971 ± 0.0095**
	**CIEDE2000 ↓**	1.0897 ± 0.1483	2.5926 ± 0.1878	0.9920 ± 0.1048	**0.8957 ± 0.1091**

**Table 2:** Quantitative comparison results (Mean ± SD) with state-of-the-art methods on the MuralDH datasets. The best results are highlighted in bold and the second-best results are underlined.

MuralDH	PSNR ↑	SSIM ↑	MAE ↓	LPIPS ↓	CIEDE2000 ↓
CMAMR	30.9226 ± 1.0631	0.9385 ± 0.0147	3.2785 ± 0.5879	0.0685 ± 0.0152	1.9588 ± 0.3067
MISF	30.3441 ± 1.1768	0.9362 ± 0.0153	4.0050 ± 0.5169	0.0753 ± 0.0137	2.1938 ± 0.3912
HINT	30.8151 ± 1.2214	0.9366 ± 0.0146	3.3635 ± 0.7633	0.0721 ± 0.0150	1.6489 ± 0.2454
ours	**31.3160** ± 1.0188	**0.9409** ± 0.0148	**3.2123** ± 0.5038	**0.0643** ± 0.1451	1.4666 ± 0.1943

**Table 3:** Quantitative results (Mean ± SD) of the ablation study on the MuralDH dataset. ✓ and ✗ indicate the presence and absence of each component, respectively.

Index	LAB Loss	Fre Loss	Metric				
			PSNR ↑	SSIM ↑	MAE ↓	LPIPS ↓	CIEDE2000 ↓
(1)	✗	✓	30.8447 ± 1.0236	0.9349 ± 0.0167	3.4732 ± 0.6216	0.0749 ± 0.1557	1.6392 ± 0.1998
(2)	✓	✗	30.7143 ± 1.0254	0.9293 ± 0.0153	3.4645 ± 0.6039	0.0675 ± 0.1602	1.5881 ± 0.2056
(3)	✓	✓	31.3160 ± 1.0188	0.9409 ± 0.0148	3.2123 ± 0.5038	0.0643 ± 0.1451	1.4666 ± 0.1943

## Data Availability

The data presented in this study are openly available in LABFNet at https://github.com/fenghualuo267/LABFNet (accessed on 10 June 2026).

## Abbreviations

The following abbreviations are used in this manuscript:

RGB	Red–Green–Blue
LABFNet	Laboratory frequency network
LAB	Laboratory
PSNR	Peak Signal-to-Noise Ratio
SSIM	Structural Similarity
MAE	Mean Absolute Error
LPIPS	Learned Perceptual Image Patch Similarity
CNN	Convolutional Neural Network
U-Net	U-shaped Network
GAN	Generative Adversarial Network
CMAMR	Contextual Mask-Aware Mural Restoration
MSE	Mean Squared Error
FDIT	frequency domain image translation
FFT	Fast Fourier Transform
MISF	Multi-level Interactive Siamese Filtering
HINT	Hypernetwork Instruction Tuning
GT	Ground truth
Fre	Frequency-domain
